# Combinational therapy with Myc decoy oligodeoxynucleotides encapsulated in nanocarrier and X-irradiation on breast cancer cells

**DOI:** 10.32604/or.2023.043576

**Published:** 2023-12-28

**Authors:** BEHROOZ JOHARI, MILAD PARVINZAD LEILAN, MAHMOUD GHARBAVI, YOUSEF MORTAZAVI, ALI SHARAFI, HAMED REZAEEJAM

**Affiliations:** 1 Department of Medical Biotechnology, School of Medicine, Zanjan University of Medical Sciences, Zanjan, Iran; 2 Zanjan Pharmaceutical Biotechnology Research Center, Zanjan University of Medical Sciences, Zanjan, Iran; 3 Nanotechnology Research Center, Ahvaz Jundishapur University of Medical Sciences, Ahvaz, Iran; 4 Department of Radiology Technology, School of Allied Medical Sciences, Zanjan University of Medical Sciences, Zanjan, Iran

**Keywords:** Combinational therapy, Antisense therapy, Myc signaling pathway, Niosomes, Radiation therapy, SeNPs

## Abstract

The Myc gene is the essential oncogene in triple-negative breast cancer (TNBC). This study investigates the synergistic effects of combining Myc decoy oligodeoxynucleotides-encapsulated niosomes-selenium hybrid nanocarriers with X-irradiation exposure on the MDA-MB-468 cell line. Decoy and scramble ODNs for Myc transcription factor were designed and synthesized based on promoter sequences of the *Bcl2* gene. The nanocarriers were synthesized by loading Myc ODNs and selenium into chitosan (Chi-Se-DEC), which was then encapsulated in niosome-nanocarriers (NISM@Chi-Se-DEC). FT-IR, DLS, FESEM, and hemolysis tests were applied to confirm its characterization and physicochemical properties. Moreover, cellular uptake, cellular toxicity, apoptosis, cell cycle, and scratch repair assays were performed to evaluate its anticancer effects on cancer cells. All anticancer assessments were repeated under X-ray irradiation conditions (fractionated 2Gy). Physicochemical characteristics of niosomes containing SeNPs and ODNs showed that it is synthesized appropriately. It revealed that the anticancer effect of NISM@Chi-Se-DEC can be significantly improved in combination with X-ray irradiation treatment. It can be concluded that NISM@Chi-Se-DEC nanocarriers have the potential as a therapeutic agent for cancer treatment, particularly in combination with radiation therapy and *in-vivo* experiments are necessary to confirm the efficacy of this nano-drug.

## Introduction

Triple-negative breast cancer is a subtype of breast cancer where cancer cells lack the human epidermal growth factor receptor 2 (HER2), estrogen receptor (ER), and progesterone receptor (PR) [1,2]. The treatment of TNBC is challenging because these cells are very invasive and cause early metastasis [3]. Common approaches to treating breast cancer include surgery, hormone therapy, chemotherapy, and radiation therapy. Despite the importance of this type of treatment, some patients do not respond efficiently to these treatments, so we need to develop effective therapies such as combination therapies [4,5]. Misregulation of proteins involved in signaling pathways such as Wnt, Hedgehog (Hh), Hippo, Notch, etc., is associated with various pathological conditions such as cancer [6–8]. Therefore, therapies based on targeting oncoproteins involved in signaling pathways can be useful for cancer treatment.

The Myc oncoprotein is a transcription factor that plays a critical role in various signaling pathways in TNBC cancer, affecting essential aspects of cancer such as cell function, growth, proliferation, and drug resistance. Therefore, it is a promising therapeutic target in TNBC therapy [9,10]. The ODNs decoy strategy is an effective approach to inhibiting transcription factors in cancer cells [11,12]. It involves the synthetic production of the cis-element of the target gene promoter in the laboratory, which is introduced into the desired cell, and competitively prevents the transcription factor from binding to downstream genes, leading to the downregulation of transcription of the downstream genes [13].

Nanocarriers can enhance drug delivery by improving drug activity and pharmacokinetics, increasing the accumulation of anticancer agents at the tumor site, and reducing side effects on normal cells. Niosomes, a type of nanocarrier, has distinct advantages for drug delivery. They are composed of nonionic surfactants and cholesterol, forming bilayer vesicles with low toxicity and proper biocompatibility. Niosomes also offer controlled drug-release properties [14].

In a study comparing niosomes prepared using the post-coating method to those synthesized with the pre-coating method, it was found that both types of niosomes exhibited a particle size range of 120–350 nm and zeta potential of +40 mV. However, the niosomes prepared by the post-coating method exhibited smaller particle sizes and demonstrated higher stability of oligonucleotides in the presence of 10% v/v serum, as compared to the niosomes synthesized through the pre-coating method [15,16]. Combining niosomes with selenium nanoparticles can potentially offer synergistic benefits in terms of drug delivery and therapeutic effects [17,18].

Combination therapy in cancer has recently received much attention, where two or more therapeutic agents or methods are used simultaneously. It can increase the effectiveness of treatment compared to a single treatment [19]. Radiotherapy is one of the current therapies that can be used for cancer treatment [20]. Furthermore, several studies have revealed that the use of X-irradiation with selenium nanoparticles (SeNPs) increases the effectiveness of irradiation by inducing DNA fragmentation, apoptosis, and activation of caspase-3 in cancer cells [21–23].

In this study, decoy ODN (DEC) against Myc TF and selenium nanoparticles (Se NP) were loaded onto chitosan polymer (Chi-Se-DEC) and encapsulated within niosome nanocarriers (NISM@Chi-Se-DEC). After the physicochemical evaluation of the nanocarriers, additional anticancer studies were performed including cell uptake, cytotoxicity, apoptosis, cell cycle, and scratch repair assays. To evaluate the anticancer effects of the NISM@Chi-Se-DEC combination with X-rays in MDA-MB-468 TNBC, all the mentioned tests were repeated under irradiation conditions (2 Gy fractionated).

## Materials and Methods

### Reagents and materials

Cholesterol (C8667; Sigma Co., USA ≥99%), bovine serum albumin (BSA) (A4612; Sigma Co., USA), sorbitan monooleate (span80, CAS.1338-43-8), polyoxyethylene sorbitan monooleate (Tween 80, CAS.9005-65-6), chloroform (Sigma Co., Germany), sodium selenite (Na_2_SeO_3_, Sigma-Aldrich, USA), MTT (57360-69-7), dulbecco’s modified eagle’s medium (DMEM), high glucose (Gibco, Carlsbad, CA, USA), trypsin-EDTA (T3924), penicillin−streptomycin (Invitrogen, Carlsbad, CA, USA), fetal bovine serum (FBS) (ES-020-B) (Sigma-Aldrich, USA), methanol, dimethyl sulfoxide (DMSO), and acetone (Emertat Co., Iran), Annexin V-FITC/PI kit (Sigma, USA), PI (Sigma−Aldrich), cell culture plates (SPL Life Sciences, South Korea), decoy and scramble ODNs (synthesized by Bioneer Inc., Daejeon, Korea), and MDA-MB-468 cell line (IBRC C10095) (purchased from the Iranian Biological Resource Center, Iran). Phosphate-buffered saline (PBS) was prepared in the laboratory.

### Cell culture

The MDA-MB-468 cancer cell line was cultured in high glucose Dulbecco’s modified Eagle’s medium (DMEM) supplemented with 10% FBS and 100 units/mL penicillin-100 µg/mL streptomycin at 37°C in a humidified atmosphere containing 5% CO_2_ in an incubator.

### Myc decoy and scrambled ODN design

A phosphorothioate (PS) modified 21-mer decoy (DEC) was synthesized based on the *Bcl2* gene, which can be targeted by Myc (Bioneer, Korea) [24]. As a negative control, scrambled ODNs (SCR) were designed by introducing mutations to the core binding site of the Myc ODN (Bioneer, Korea). Each of the forward and reverse strands of DEC and SCR ODNs was dissolved in sterile TE (Tris/EDTA) buffer, annealed at 95°C for 10 min, and then cooled at room temperature to obtain double-stranded ODNs [25]. The obtained ODNs were quantified using nanodrop spectrophotometry. To trace cell uptake, Cy3-labeled ODNs were also designed. The sequence of the designed ODNs is shown below, where the core binding site is shown in boldface, * indicates PS modifications and underlining/italics indicate mutations:

Decoy sequences:

F [5′ T*TGGCAC**CACGTG**GTGGCGA*G 3′]

R [5′ A*ACCGTGGTGCA*C*CACCGCT*C 3′]

SCR sequences:

F [5′ T*TGGCAC*A*A*AT*TGGTGGCGA*G 3′]

R [5′ A*ACCGTGTTTAACCACCGCT*C 3′]

### Synthesis of nanocarriers

Preparation of lipid-thin film: The lipid-thin film was prepared by synthesizing niosomes using the thin layer hydration method with some modifications. The process involved mixing 6% w/v of each of the Tween 80 and Span 80 surfactants in a 1:1 weight ratio, adding 1.6% w/v of cholesterol, and dissolving it in 2 mL of organic solvent. The mixture was then placed in a rotary evaporator at 150 rpm and 60°C for 20 min to evaporate the organic solvent and form a lipid-thin film, which was then placed in a vacuum oven for 2 h.

Free SeNPs synthesis: Selenium nanoparticles (SeNPs) were synthesized using the green synthesis method. Briefly, 2% w/v of bovine serum albumin (BSA) was dissolved in distilled water, and then 1% w/v of sodium selenite (Na_2_SeO_3_) was added and stirred for 45 min at 100°C and 150 rpm. The change in the solution’s color to red-orange was used as an indicator of the formation of SeNPs. The colloidal SeNPs were purified by dialysis using a 12-kDa molecular weight cutoff against ultrapure water overnight to remove excess BSA, Na_2_SeO_3_ salt, and other precursors.

Free NISM & NISM@Chi: To form NISM, an appropriate volume of distilled water was used to hydrate the thin lipid layer. Conversely, a solution consisting of distilled water and 0.2% w/v chitosan was utilized to formulate chitosan-coated niosomes, referred to as NISM@Chi. All samples were synthesized under sonication conditions and kept at stirred conditions.

NISM@Chi-Se: To prepare NISM@Chi hybridized SeNPs (NISM@Chi-Se), an appropriate volume of distilled water with 0.2% w/v chitosan and 0.02% w/v SeNPs was added dropwise into the thin lipid layer under stirrer and sonication conditions. The obtained sample was kept at stirred conditions.

ODNs Encapsulated Nanocarriers: To synthesize SCR or DEC encapsulated NISM@Chi (NISM@Chi-SCR or NISM@Chi-DEC), an aqueous phase containing 0.2% w/v chitosan and 3.124% v/v SCR or 3.36% v/v DEC was kept under constant stirring at room temperature. After 2 h, the solution was added dropwise into the thin lipid layer under stirrer and sonication conditions. The resulting solution was incubated under the same conditions for 2 h. The same method was used to create the final formulations (NISM@Chi-Se-SCR or NISM@Chi-Se-DEC). The aqueous phase contained 0.2% w/v chitosan, 0.02% w/v SeNPs, and 3.124% v/v SCR or 3.36% v/v DEC.

### Physicochemical characterization

The physicochemical properties of the NISM nanocarriers were determined using Fourier Transform Infrared Spectroscopy (FTIR), Dynamic Light Scattering (DLS), and Field Emission Scanning Electron Microscope (FESEM). Fourier IR spectroscopy (Bruker, Tensor 27, Biotage, Germany) to identify the chemical structure and composition of Chi, Se, DEC, SCR, NISM, NISM@Chi, NISM@Chi-Se, NISM@Chi-SCR, NISM@Chi-DEC, NISM@Chi-Se-SCR, and NISM@Chi-Se-DEC. All samples were mixed with potassium bromide at a 1:20 ratio and then pelleted at a pressure of 10 N.

The average hydrodynamic diameter (Z-average), polydispersity index (PDI), and surface charge (*zeta* potential) of the NISM nanocarriers were determined using DLS (Malvern Instruments, Worcestershire, UK, Nano ZS model). For DLS analysis, 200 μL of each sample was diluted in 2 mL of deionized water in a clean Malvern sample tube until the absorbance at 633 nm was 0.07 ± 0.02 units.

FESEM was used to determine the size and morphology of the NISM nanocarriers. The dried sample was covered with gold and observed at 100,000× magnification and an accelerating voltage of 15 kV using FESEM (MIRA TESCAN, Czech Republic).

Physical stability tests were performed on NISM@B-SCR and NISM@B-DEC to investigate mean hydrodynamic diameter (Z-average) and decoy ODN leaching in NISM@Chi nanocarriers during storage. NISM@Chi-Se-SCR and NISM@Chi-Se-DEC were freshly prepared and stored at 4 ± 2°C and room temperature for 60 days [26].

The entrapment efficiency (EE%) of ODN decoy (DEC) in niosomes nanocarriers was calculated using the centrifugation method [27]. Briefly, NISM nanocarriers were diluted in phosphate buffer (pH 7.4) and centrifuged at 14,000 rpm for 30 min to collect free ODNs. This process was repeated five times to ensure that all free ODNs had been collected. The nanodrop spectrophotometer was used to quantify non-entrapment ODN decoy at a 260 nm wavelength. As shown below, decoy ODN entrapment efficiency (%EE) was calculated:

$$\begin{array}{l} \;\%\;Entrapment\;Efficiency\;\left(\%EE\right)\\ \;\;=\;\frac{Total\;decoy\;ODNs-Free\;decoy\;ODNs}{Total\;decoy\;ODNs}\times 100 \end{array}$$

### ODN release study

The release behavior of ODNs in NISM nanocarriers was assessed according to our previous report [27]. Briefly, to perform a release study, purified NISM@Chi-SCR, NISM@Chi-DEC, NISM@Chi-Se-SCR, and NISM@Chi-Se-DEC (without free ODNs) were diluted in 1.5 mL of phosphate buffer (PBS, pH = 5.8 and pH = 7.4) and then placed in an incubator shaker at 120 rpm and 37°C. The release media were collected and replaced with an equal volume of fresh PBS. Finally, the collected media were quantified using a nanodrop spectrophotometer at a 260 nm wavelength.

### Hemocompatibility assay

To assess the hemocompatibility of NISM nanocarriers and their effect on red blood cells (RBCs), a hemolysis assay was conducted. Healthy human blood was collected and prepared by diluting the RBC pellet with cold PBS. This study was approved by the Ethics Committee of Zanjan University of Medical Sciences (Ethical Code: IR.ZUMS.REC.1399.316). The nanocarriers were prepared at various concentrations and added to the RBC samples, with PBS and SDS serving as negative and positive controls, respectively. The samples were then incubated, and the absorbance of hemoglobin was measured to determine the extent of RBC membrane damage and hemolysis caused by the nanocarriers [28]. This experiment was repeated three times, and the percentage of hemolysis was calculated as:

$$Hemolysis\%=\frac{A\;treated\;sample-A\;negative\;control}{A\;posetive\;control-A\;negative\;control}\times 100$$

In this equation, A treated sample, A negative control, and A positive control are representative of the mean absorbance of the sample, negative control, and positive control, respectively.

### Cellular uptake assay of nanocarriers

To evaluate the cell uptake efficiency of NISM nanocarriers in MDA-MB-468 cells, we used Cy3-labeled ODNs and flow cytometry, based on our previous study [29]. Briefly, 3 × 10^4^ cells were seeded in a 24-well plate and incubated until approximately 70% confluency. Next, the medium was removed. The cells were washed with PBS. Subsequently, three concentrations of NISM@Chi-Se-Cy3-labeled ODNs (0.25, 0.5, and 1 μg/mL) were prepared, while 1 mg/mL of NISM@Chi-Se was used as a blank nanocarrier control. After 6 h, the total treatment media was removed, and 500 μL of fresh complete medium (10% FBS, 89% DMEM, and 1% pen-strep) was added to all cell groups. After 24 h (post-treatment), the cells were harvested from each group and analyzed using flow cytometry (BD Biosciences, San Jose, CA, USA) and FlowJo v7 software (Tree Star, Ashland, OR, USA).

### Cellular toxicity assay

The effect of nanocarriers on the cytotoxicity of MDA-MB-468 cells was investigated using the MTT assay, with some modifications based on our previous study [30]. Briefly, 1.5 × 10^4^ MDA-MB-468 cells were seeded in a 96-well plate and incubated until they reached approximately 70%–80% confluency. The cells were then treated with different concentrations (0.5, 1, 2, and 4 μg/mL) of various nanocarriers (NISM, SeNPs, NISM@Chi, NISM@Chi-SCR, NISM@Chi-DEC, NISM@Chi-Se, NISM@Chi-Se-SCR, and NISM@Chi-Se-DEC). After 6 h of treatment, the treatment medium was completely removed, and 500 μL of fresh medium was added to all wells. The cells were then exposed to a 2 Gy fractionated X-ray. 24 h post-treatment, the media from cell groups were removed, and 20 μL of MTT solution (5 mg/mL) was added to each well. After removing the MTT solution, the cell pellets were dissolved in 100 μL of DMSO per well. Finally, the absorbance was measured at 570/630 nm using an ELISA reader. All aforementioned steps were also performed without X-ray exposure.

### Cell cycle assay

The effect of nanocarriers on the cell cycle of MDA-MB-468 cells, both with and without irradiation, was evaluated using flow cytometry based on our previous study [25]. Briefly, 5 × 10^4^ cells were seeded in a 12-well plate and incubated under standard conditions. When the cells reached approximately 70% confluency, they were treated with 1 μg/mL of different nanocarriers (NISM@Chi-Se, NISM@Chi-SCR, NISM@Chi-DEC, NISM@Chi-Se-SCR, and NISM@Chi-Se-DEC). 6 h post-treatment, the medium from all cell groups were removed, and 500 μL of fresh complete medium was added to each well. The plate of cells was then exposed to a 2 Gy fractionated X-ray. After 24 h, the cells were harvested and pelleted by centrifugation at 1200 rpm for 3 min. The cell pellets were washed with 50 μL of PBS and fixed with 70% cold ethanol. Extra ethanol was removed by centrifugation. Finally, the cells were treated with 1 mL of PI Master MIX solution (950 μL of PBS, 40 μL of PI, and 10 μL of RNase) and incubated at room temperature for 30 min. The cells were then analyzed using flow cytometry (BD Biosciences, San Jose, CA, USA), and the data were analyzed using FlowJo v7 software (Tree Star, Ashland, OR, USA) to determine the cell population percentage in each phase of the cell cycle. All aforementioned steps were also performed without X-ray exposure.

### Apoptosis assay

To evaluate the apoptosis rate in MDA-MB-468 cells (5 × 10^4^ cells/12-well plate) 24 h after treatment with 1 μg/mL of various nanocarriers (NISM@Chi-Se, NISM@Chi-SCR, NISM@Chi-DEC, NISM@Chi-Se-SCR, and NISM@Chi-Se-DEC), flow cytometry was used as described in our previous study [25]. Briefly, 6 h after treatment, the cells were washed with fresh complete medium and exposed to a 2 Gy fractionated X-ray. The cells were then harvested, washed, and stained with Annexin V-FITC and PI according to the manufacturer’s protocol. Finally, flow cytometry data were recorded using BD Biosciences equipment (San Jose, CA, USA) and analyzed with FlowJo v.7 software (Tree Star, Ashland, OR, USA). Additionally, all aforementioned steps were performed without X-ray exposure to serve as a control.

### Wound-healing (Scratch) assay

To investigate the cell migration inhibition rate (%) in MDA-MB-468 cells, the scratch assay was performed as previously reported [25]. Briefly, 6 × 10^4^ cells were seeded in 12-well plates and incubated under standard conditions for 24 h. When the cell confluency reached approximately 70%, an artificial scratch was created in the center of the plate using a 10 μL pipette tip. After washing with PBS, all cell groups were photographed 0 h. The cells were then treated with 1 μg/mL of various nanocarriers (NISM@Chi-Se, NISM@Chi-Se-SCR, and NISM@Chi-Se-DEC). Six hours post-treatment, the cells were exposed to a 2 Gy fractionated X-ray. Finally, the cell plate was photographed at 96 h after treatment, and the migration inhibition rate (%) was measured using ImageJ software (1.52a). Additionally, all aforementioned steps were performed without X-ray exposure to serve as a control.

### Statistical analysis

The data obtained from the experiments were analyzed using Graphpad Prism 6 software and expressed as mean ± standard deviation. Each experiment was repeated at least three times to ensure reproducibility and accuracy of the results. One-way or two-way analysis of variance (ANOVA) was used as a statistical test to determine the significance of differences between experimental groups. A value of *p* < 0.05 was considered statistically significant. The level of significance was indicated by asterisks, with * representing *p* < 0.05, **p < 0.01, ***p < 0.001, and ****p < 0.0001.

## Results

### Physicochemical characterization

**Figure 1:**
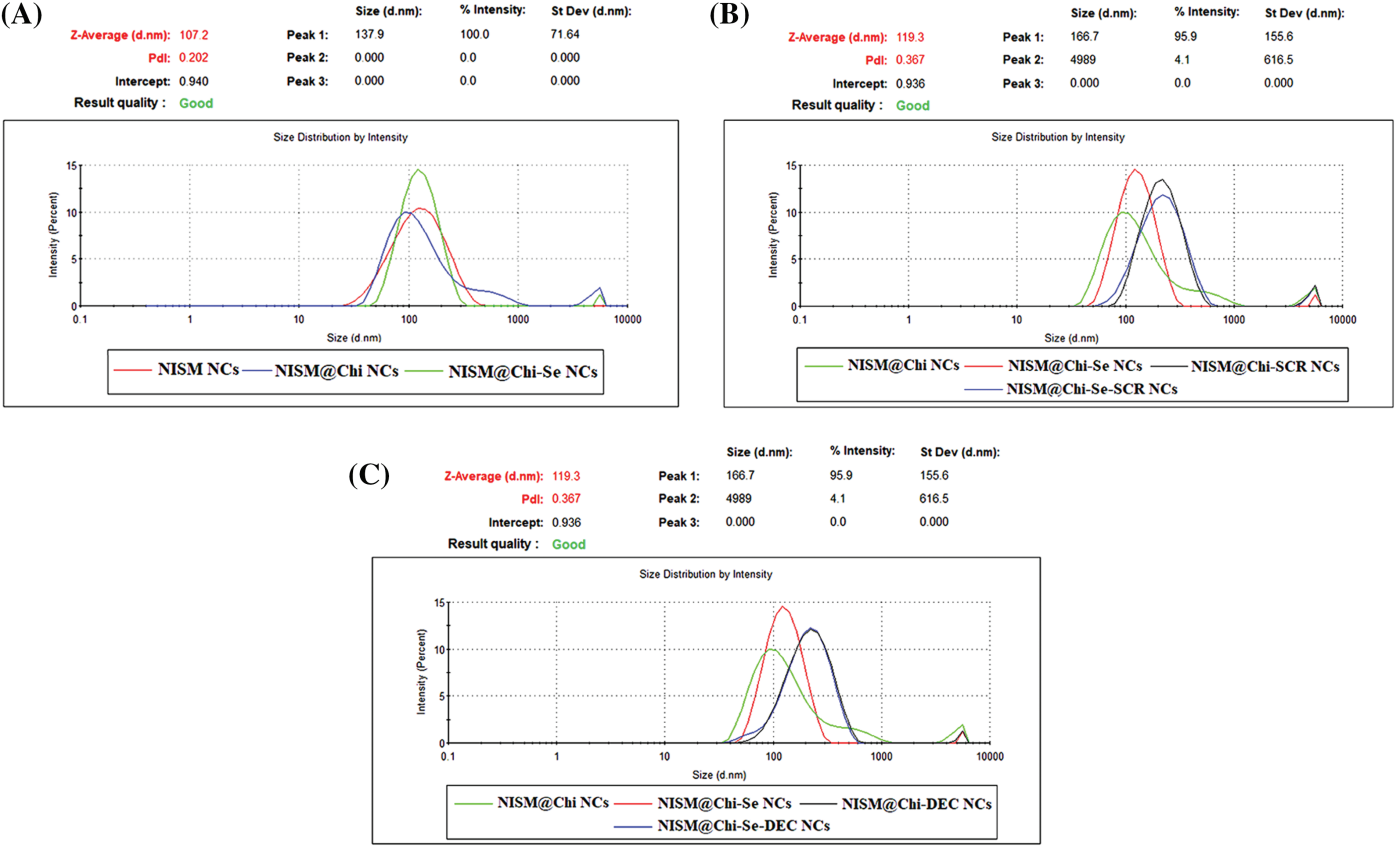
Average size and average PDI for groups of NISM, NISM@Chi, NISM@Chi-SCR, NISM@Chi-DEC, NISM@Chi-Se, NISM@Chi-Se-SCR, NISM@Chi-Se-DEC.

**Figure 2:**
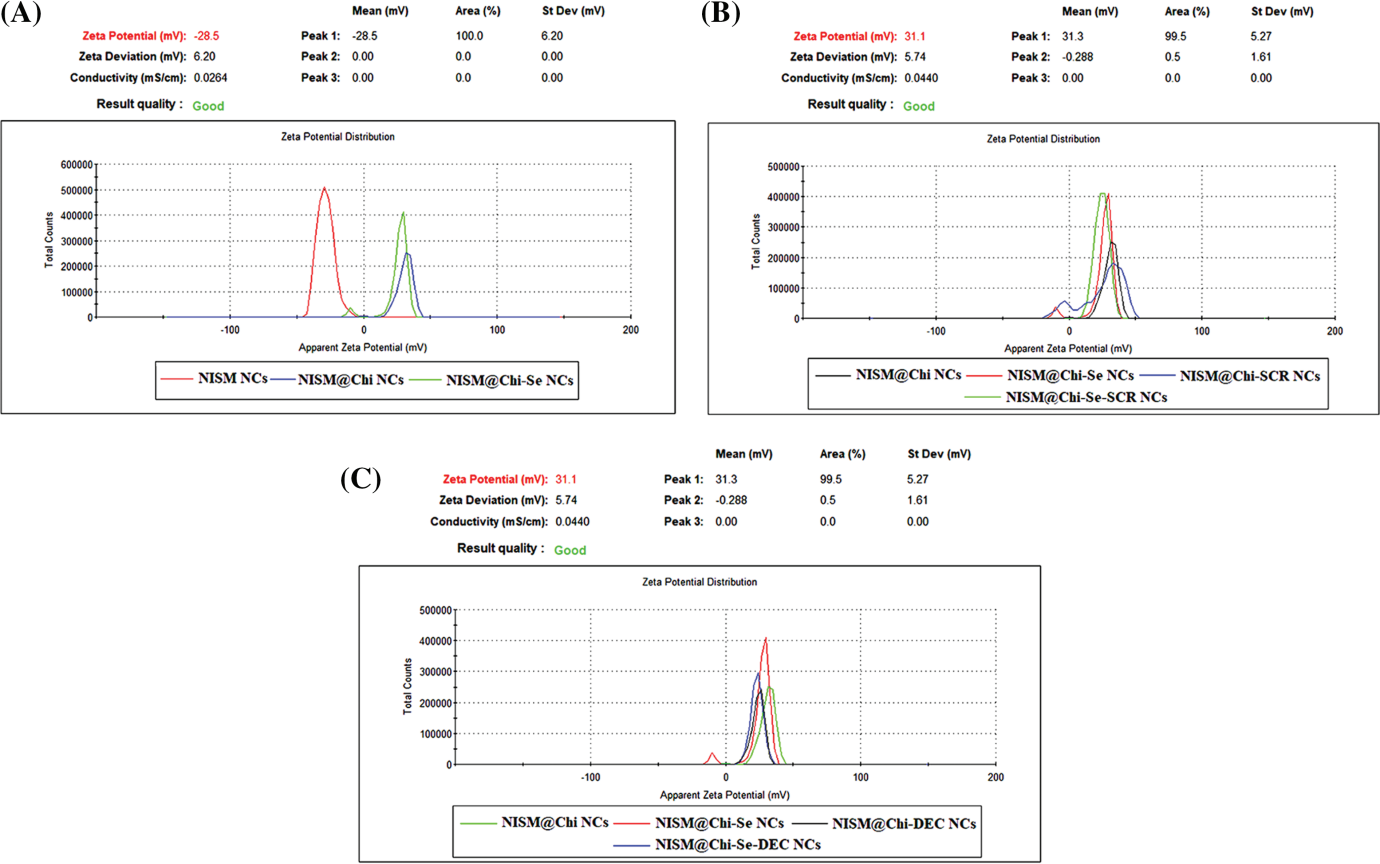
Average zeta potential of nanoparticles NISM, NISM@Chi, NISM@Chi-Se, NISM@Chi-SCR, NISM@Chi-DEC, NISM@Chi-Se-SCR, and NISM@Chi-Se-DEC.

Physicochemical properties of nanocarriers, such as surface charge, size, polydispersity index (PDI), morphology, release behavior of ODNs, and safety were analyzed. The average hydrodynamic size, zeta potential, and PDI were determined using DLS and are presented in Figs. 1 and 2. The sizes of the NISM, NISM@Chi, NISM@Chi-Se, NISM@Chi-SCR, NISM@Chi-DEC, NISM@Chi-Se-SCR, and NISM@Chi-Se-DEC nanocarriers were 108.06, 120.9, 122.23, 220.56, 202.3, 227.6, and 229 nm, respectively. The PDIs for these groups were 0.195, 0.382, 0.366, 0.379, 0.362, 0.344, and 0.372, respectively.

*Zeta* potential was determined using DLS, and the results are presented in Fig. 2. The average *zeta* potentials for NISM, NISM@Chi, NISM@Chi-Se, NISM@Chi-SCR, NISM@Chi-DEC, NISM@Chi-Se-SCR, and NISM@Chi-Se-DEC were −25.09, +29.7, +28.7, +25.26, +25.4, +25.06, and +23.53 mV, respectively.

**Figure 3:**
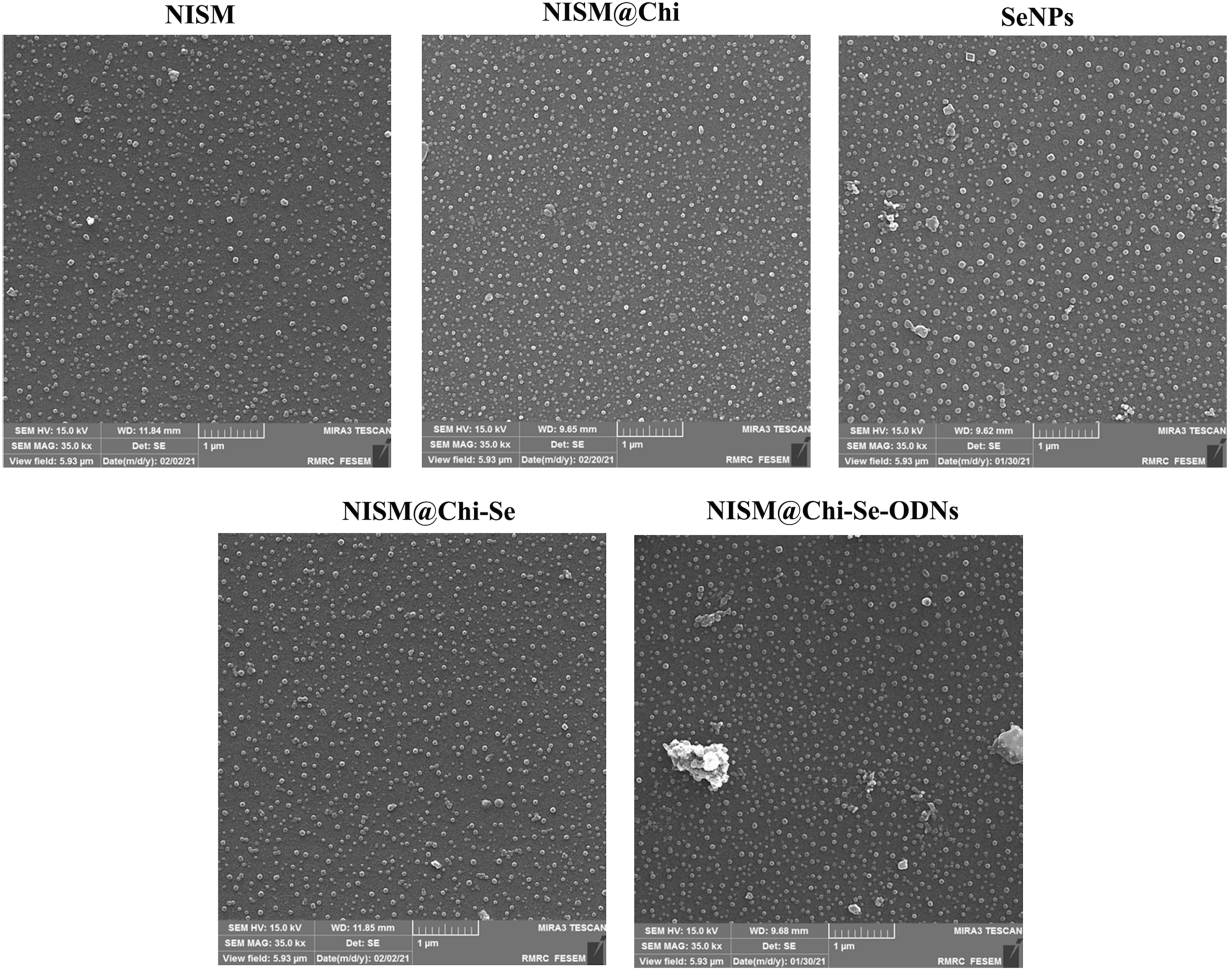
FESEM image of NISM, NISM@Chi, SeNPs, NISM@Chi-Se, NISM@Chi-Se-ODNs nanosystems.

The morphology of the nanocarriers was examined using FESEM, and the results are presented in Fig. 3. The average sizes of NISM, NISM@Chi, NISM@Chi-Se, NISM@Chi-Se-DEC, and SeNP were 46.54, 51.25, 54.22, 59.75, and 89.81 nm, respectively.

**Figure 4:**
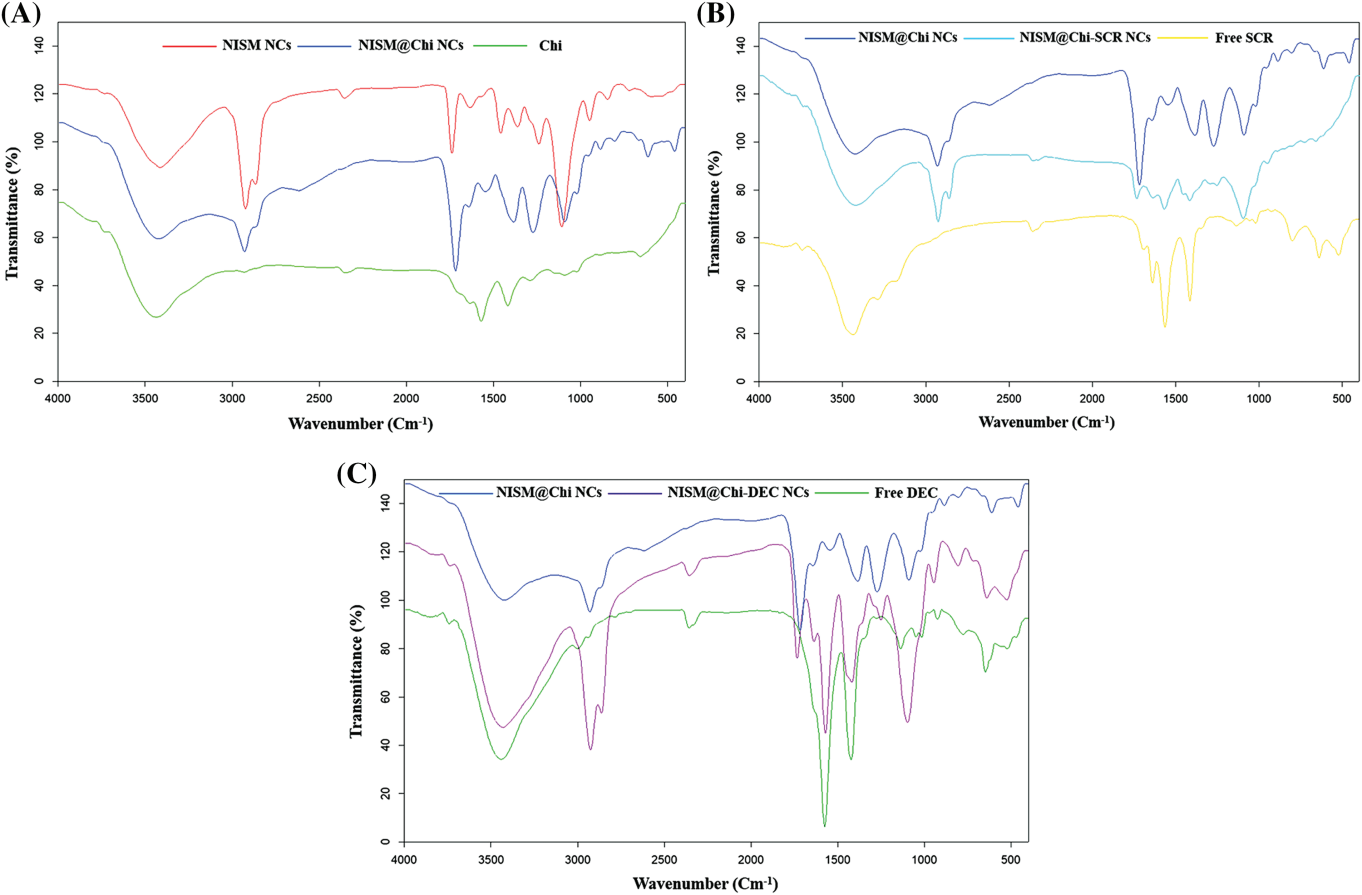
FT-IR diagram for NISM@Chi, NISM@Chi-SCR, and NISM@Chi-DEC groups.

Fig. 4A displays the FT-IR spectrum of the NISM@Chi nanocarrier, which shows peaks at 1700 and 3400 cm^−1^ corresponding to the NISM nanocarrier and peaks at 3291 and 3361 cm^−1^ corresponding to chitosan (Chi). The presence of these peaks in the NISM@Chi spectrum provides evidence of the successful synthesis of this nanocarrier.

Figs. 4B and 4C show the FT-IR spectra of the NISM@Chi-SCR and NISM@Chi-DEC nanocarriers, respectively. In Fig. 4B, the SCR exhibits a peak at 1650 cm^−1^. The presence of peaks corresponding to chitosan, NISM, and SCR indicates successful encapsulation of the payloads. In Fig. 4C, the oligodeoxynucleotide (DEC) exhibits a peak at 3440 cm^−1^, which is visible in the NISM@Chi-DEC spectrum. Additionally, the peaks of chitosan and NISM can also be observed in this spectrum, supporting the evidence of successful synthesis.

**Figure 5:**
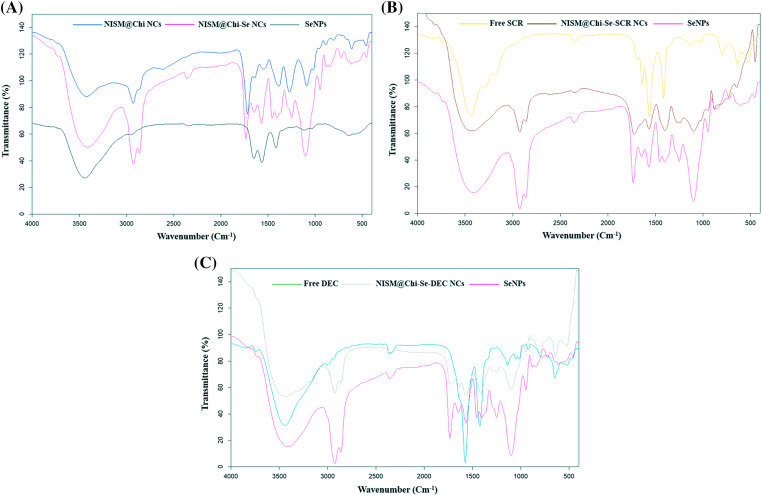
FT-IR diagram for NISM@Chi-Se, NISM@Chi-Se-SCR, and NISM@Chi-Se-DEC.

Fig. 5A shows the FT-IR spectrum of the NISM@Chi-Se nanocarrier, and the presence of peaks at 1749, 1395, and 1580 cm^−1^ indicates the presence of selenium (Se). Additionally, the presence of peaks corresponding to chitosan and NISM in the spectrum provides further evidence of the successful synthesis of this nanocarrier.

Figs. 5B and 5C show the FT-IR spectra of the NISM@Chi-Se-SCR and NISM@Chi-Se-DEC nanocarriers, respectively. In Fig. 5B, the presence of peaks corresponding to selenium at 1749, 1395, and 1580 cm^−1^, as well as the SCR peak at 1650 cm^−1^, and the presence of chitosan and NISM peaks provide evidence of the successful synthesis of the NISM@Chi-Se-SCR nanocarrier. In Fig. 5C, the presence of peaks corresponding to selenium and DEC, as well as the peaks of chitosan and NISM, provide evidence of the successful synthesis of the NISM@Chi-Se-DEC nanocarrier.

The encapsulation percentage of the NISM@Chi-Se-DEC nanocarriers was determined using the centrifuge method. The experiment was repeated five times, and the obtained encapsulation percentages were 80.94, 80.89, 81.05, 82.41, and 81.56. The average encapsulation efficiency (EE%) was calculated to be 81.37%, with a standard deviation of 0.638.

**Figure 6:**
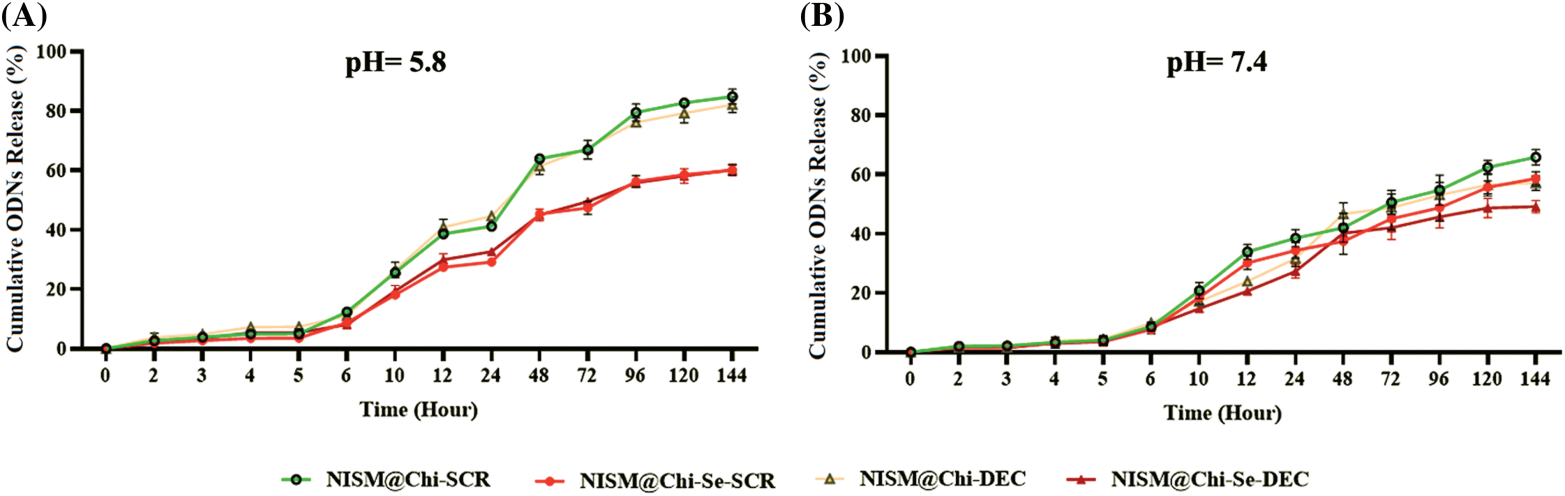
Evaluation of oligodeoxynucleotide release from NISM@Chi-SCR, NISM@Chi-DEC, NISM@Chi-Se-SCR, and NISM@Chi-Se-DEC nanocarriers at (A) pH = 5.8 and (B) pH = 7.4.

To investigate the release behavior of the nanocarriers, a study was performed using PBS media at 37°C with pH values of 7.4 and 5.8. Fig. 6A shows the release of oligodeoxynucleotides (ODNs) from the niosomal nanocarriers at pH = 5.8. Approximately 80% of the ODNs in NISM@Chi-SCR and NISM@Chi-DEC were released within 144 h, while NISM@Chi-Se-SCR and NISM@Chi-Se-DEC showed about 60% release of ODNs within the same time.

Fig. 6B shows the release of ODNs at pH = 7.4 for the various groups. After 144 h, the release from NISM@Chi-SCR, NISM@Chi-DEC, NISM@Chi-Se-SCR, and NISM@Chi-Se-DEC was found to be 65%, 56%, 60%, and 50%, respectively.

### Hemolysis assay revealed the good hemocompatibility of NPs

To analyze the blood biocompatibility of the nanocarriers, a hemolysis assay was performed by incubating different groups of nanocarriers with purified red blood cells (RBCs) and calculating the percentage of hemolysis. Suppl. Fig. S1 shows the hemolysis percentages of different groups of nanocarriers. The hemolysis percentage of NISM@Chi-Se-DEC at concentrations of 6.25, 12.5, 25, 50, 100, and 200 mg/mL was found to be 2%, 3%, 4%, 6%, 8%, and 11%, respectively.

### Nanocarriers enhanced cellular uptake of Cy3-labeled ODNs

**Figure 7:**
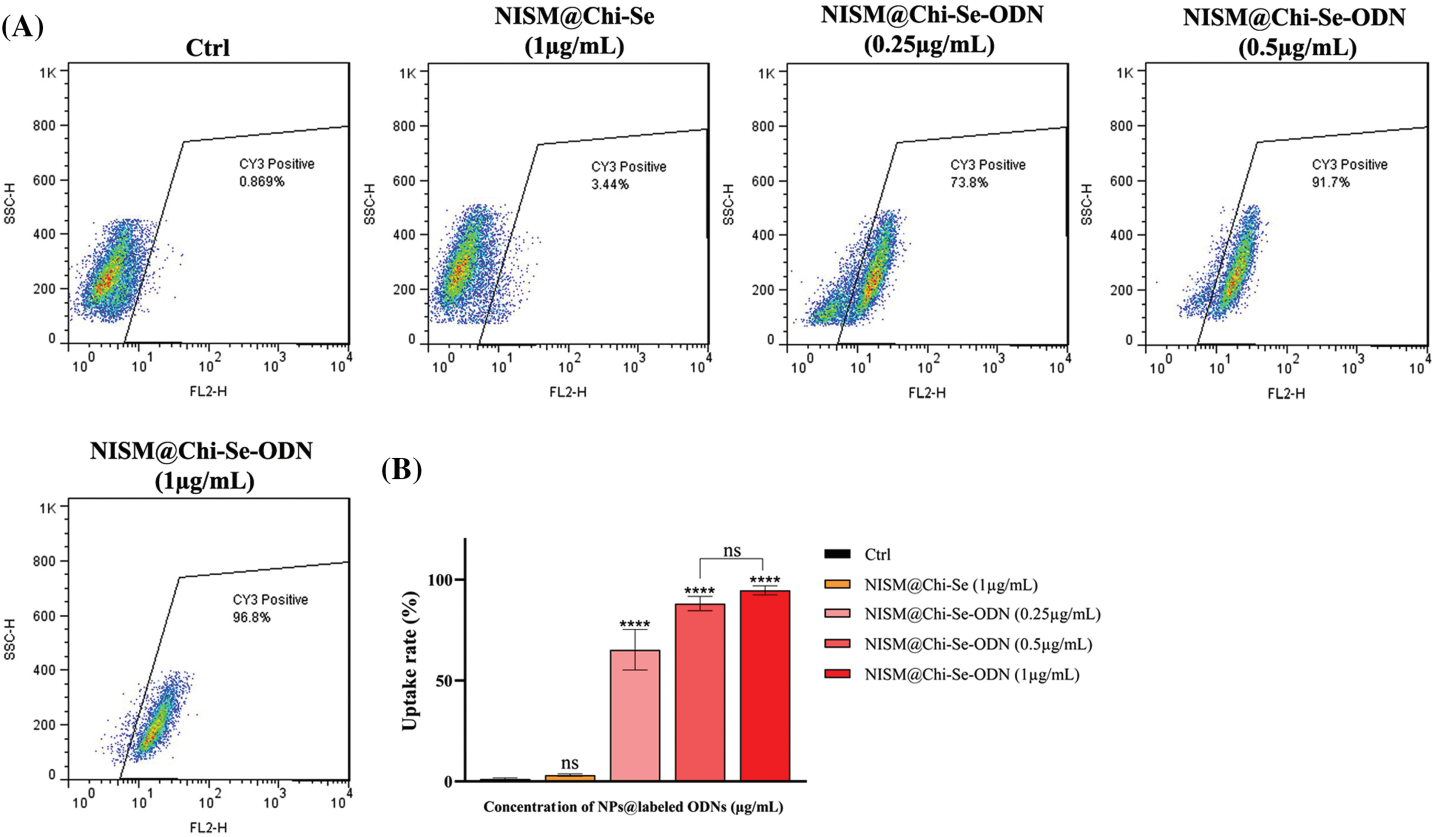
(A) Uptake analysis of NISM@Chi-Se-ODNs (Cy3-labeled ODNs) into MDA-MB-468 cells at concentrations of 0.25, 0.5, and 1 μg/mL. (B) Graph of the statistical analysis of cell uptake rate. Not significant (ns) and ****p < 0.0001.

To evaluate the efficiency of nanoparticle entrance into cells, a cell uptake assay was performed using flow cytometry. As shown in Fig. 7, the amount of cell uptake in the control group (without any treatment) and NISM@Chi-Se (as a negative control for nanocarriers) at a concentration of 1 mg/mL was low (0.869% and 3.44%, respectively). This suggests that the uptake rate of NISM@Chi-Se nanocarriers into the cells was not significant compared to the control group.

However, at concentrations of 0.25, 0.5, and 1 μg/mL of NISM@Chi-Se-ODNs (ODNs labeled with Cy3), cellular uptake was significantly increased (73.8%, 91.7%, and 96.8%, respectively). The entry rate of NISM@Chi-Se-ODNs into the cells at all concentrations used showed a significant difference compared to the control group.

### Cellular toxicity evaluation with and without X-irradiation exposure

**Figure 8:**
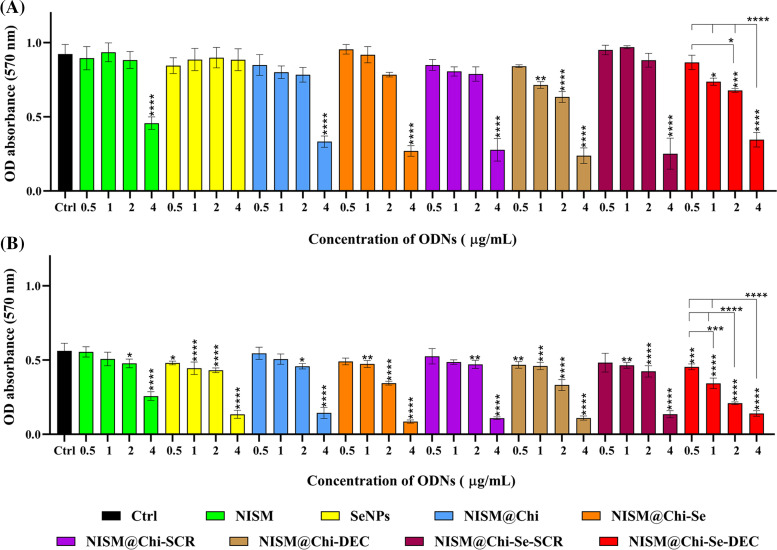
Evaluation of cytotoxicity after treatment with NISM, SeNPs, NISM@Chi, NISM@Chi-SCR, NISM@Chi-DEC, NISM@Chi-Se, NISM@Chi-Se-SCR, NISM@Chi-Se-DEC on MDA-MB-468 cells at concentrations of 0.5, 1, 2 and 4 μg/mL in the condition of (A) without X-irradiation and (B) under X-irradiation. *p < 0.05, **p < 0. 01, ***p < 0.001, and ****p < 0.0001.

As Fig. 8A shows the cytotoxicity results of the nanocarriers without X-irradiation exposure. Treatment with 0.5 μg/mL of all nanocarrier groups did not significantly affect cytotoxicity compared to the control group (cells without treatment, Ctrl). The SeNPs group did not significantly increase cytotoxicity at any of the tested concentrations.

Cytotoxicity promotion in the groups treated with NISM, NISM@Chi, NISM@Chi-Se, NISM@Chi-SCR, and NISM@Chi-Se-SCR nanocarriers at concentrations of 0.5, 1, and 2 μg/mL was not significantly different compared to the control group. However, NISM@Chi-DEC and NISM@Chi-Se-DEC nanocarriers at all used concentrations, except 0.5 μg/mL, significantly increased cytotoxicity in comparison with the control group. All nanocarrier groups, except SeNPs, at a concentration of 4 μg/mL caused a significant increase in cytotoxicity compared to the control group.

Fig. 8B shows the cytotoxicity results of the nanocarriers under X-irradiation conditions. All nanocarrier groups used at concentrations of 2 and 4 μg/mL led to a significant increase in cytotoxicity compared to the control group (cells without treatment, Ctrl). Treated groups with SeNPs, NISM@Chi-DEC, and NISM@Chi-Se-DEC nanocarriers at concentrations of 0.5 and 1 μg/mL resulted in a significant increase in cytotoxicity compared to the control group. NISM@Chi-Se nanocarriers at a concentration of 1 μg/mL caused an increase in cytotoxicity compared to the NISM and NISM@Chi groups, which could be due to the presence of selenium in the NISM@Chi-Se formulation.

### Cell cycle evaluation without X-irradiation exposure

Suppl. Fig. S2 shows the results of cell cycle arrest without X-irradiation exposure. After 24-h treatment with culture medium (Ctrl), NISM@Chi-Se, NISM@Chi-SCR, NISM@Chi-DEC, NISM@Chi-Se-SCR, and NISM@Chi-Se-DEC, the percentages of cell cycle arrest in the G1 phase were 63.77%, 65.38%, 66.24%, 74.47%, 64.24%, and 70.80%, respectively. Although the NISM@Chi-DEC nanocarrier group induced slightly higher G1 arrest than the NISM@Chi-Se-DEC group, the difference was not statistically significant. The percentage of cell population arrested in the G1 phase in the treated groups with NISM@Chi-DEC and NISM@Chi-Se-DEC nanocarriers was significantly higher than in the other groups.

### Cell cycle evaluation under X-irradiation conditions

**Figure 9:**
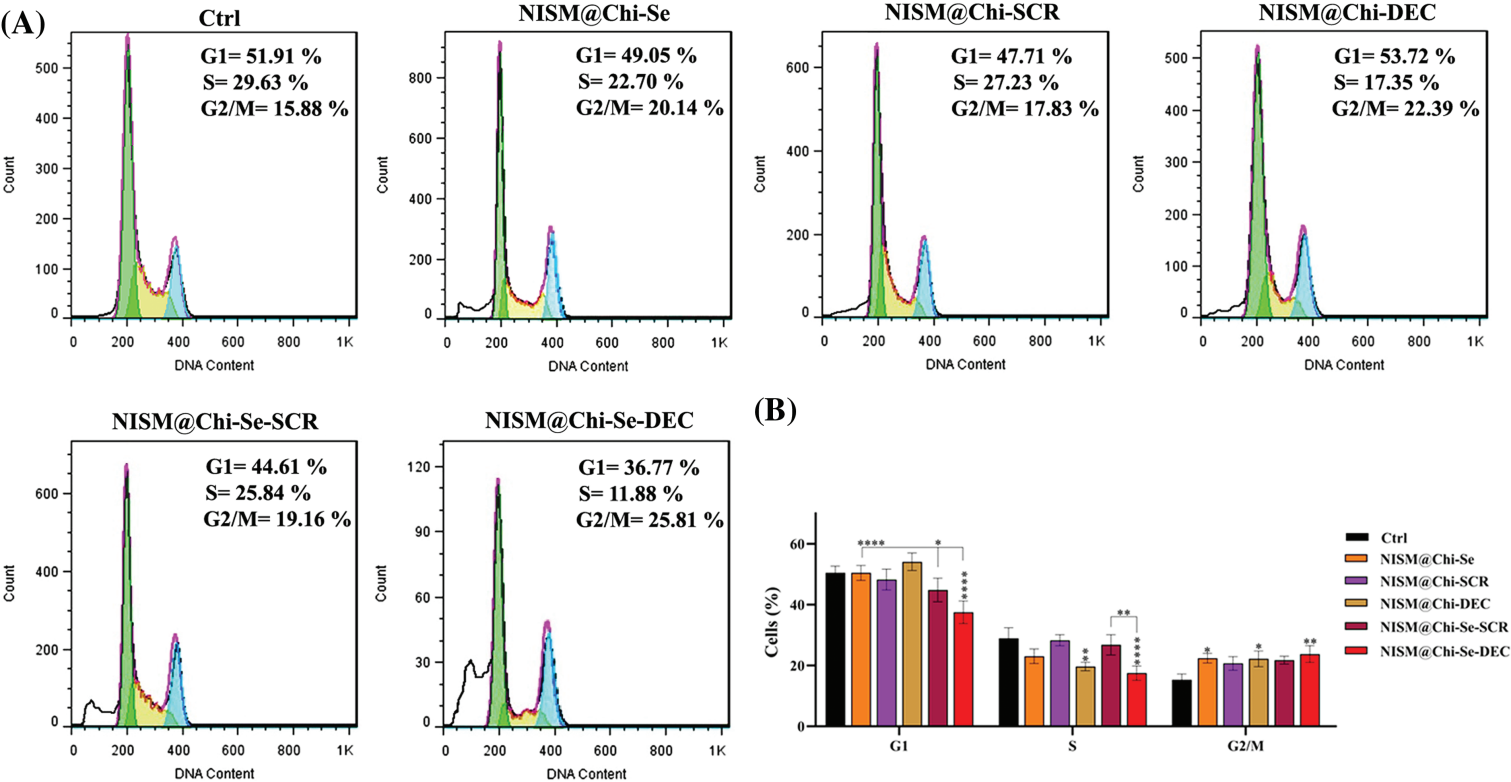
(A) Evaluation of cell population distribution in the cell cycle of MDA-MB-468 cells after treatment with NISM@Chi-Se, NISM@Chi-SCR, NISM@Chi-DEC, NISM@Chi-Se-SCR, and NISM@Chi-Se-DEC with a concentration of 1 μg/mL in under X-irradiation conditions. (B) Statistical analysis graph. *p < 0.05, **p < 0. 01, and ****p < 0.0001.

Fig. 9 shows the results of cell cycle arrest under X-irradiation conditions. After 24-h treatment with culture medium (Ctrl), NISM@Chi-Se, NISM@Chi-SCR, NISM@Chi-DEC, NISM@Chi-Se-SCR, and NISM@Chi-Se-DEC, the percentages of cell cycle arrest in the G2/M phase were 15.88%, 20.14%, 17.83%, 22.39%, 19.16%, and 25.81%, respectively. The cell arrest percentage at the G2/M phase in the treated group with NISM@Chi-Se-DEC was significantly higher than in the control. Additionally, the Sub-G1 phase was observed in cell groups treated with NISM@Chi-Se, NISM@Chi-Se-SCR, and NISM@Chi-Se-DEC nanocarriers, which could indicate the amount of cell apoptosis in each group.

### Assessment of apoptosis without X-irradiation exposure

Suppl. Fig. S3 shows the results of apoptosis induction without X-irradiation exposure. After treatment with culture medium (Ctrl), NISM@Chi-Se, NISM@Chi-SCR, NISM@Chi-DEC, NISM@Chi-Se-SCR, and NISM@Chi-Se-DEC nanocarriers, the percentage of cells undergoing apoptosis was 6.43%, 8.16%, 10.02%, 15.2%, 8.61%, and 14.66%, respectively. Additionally, the amount of cell apoptosis induced by the NISM@Chi-DEC nanocarrier group was slightly higher than the NISM@Chi-Se-DEC group, but the difference was not statistically significant. The percentage of induced apoptosis in the NISM@Chi-DEC and NISM@Chi-Se-DEC groups was significantly higher than in the other groups.

### Assessment of apoptosis under X-irradiation conditions

**Figure 10:**
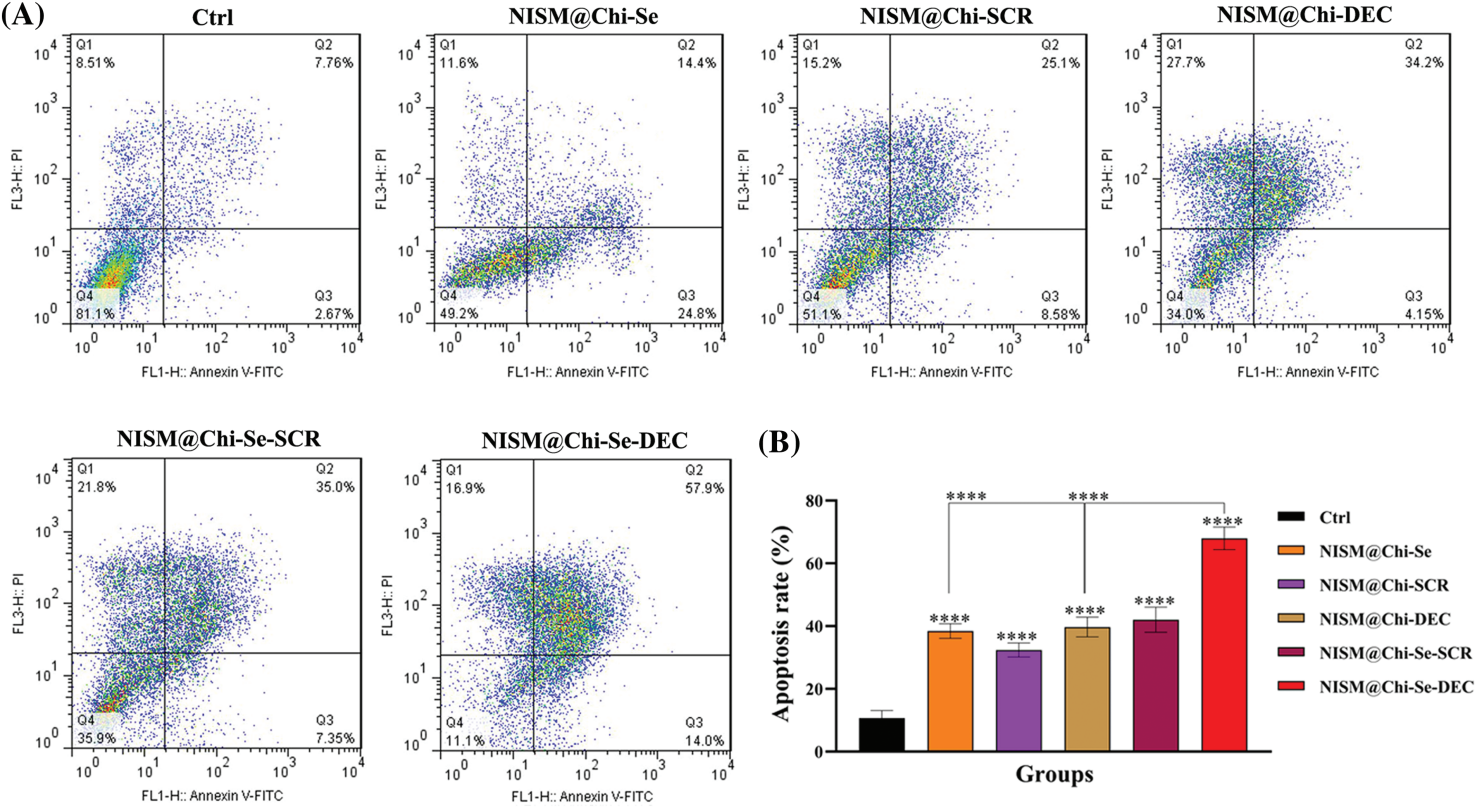
(A) Apoptosis evaluation under X-irradiation condition in MDA-MB-468 cells 24 h after treatment with culture medium (Ctrl), NISM@Chi-Se, NISM@Chi-SCR, NISM@Chi-DEC, NISM@Chi-Se-SCR and NISM@Chi-Se-DEC groups with a concentration of 1 μg/mL. (B) The column graph shows the apoptosis rate analysis following nanocarriers and/or X-irradiation treatment. ****p < 0.0001. Q1: Necrosis; Q2: Late apoptosis; Q3: Early apoptosis; Q4: Live cells.

Fig. 10 shows the results of apoptosis induction under X-irradiation conditions. After 24-h treatment with culture medium (Ctrl), NISM@Chi-Se, NISM@Chi-SCR, NISM@Chi-DEC, NISM@Chi-Se-SCR, and NISM@Chi-Se-DEC nanocarriers, the percentage of cells undergoing apoptosis was 10.43%, 39.2%, 33.68%, 38.35%, 42.35%, and 71.9%, respectively. The percentage of induced apoptosis in the NISM@Chi-Se-DEC group was significantly higher than in the other groups.

### Cell migration inhibition analysis with and without X-irradiation exposure

**Figure 11:**
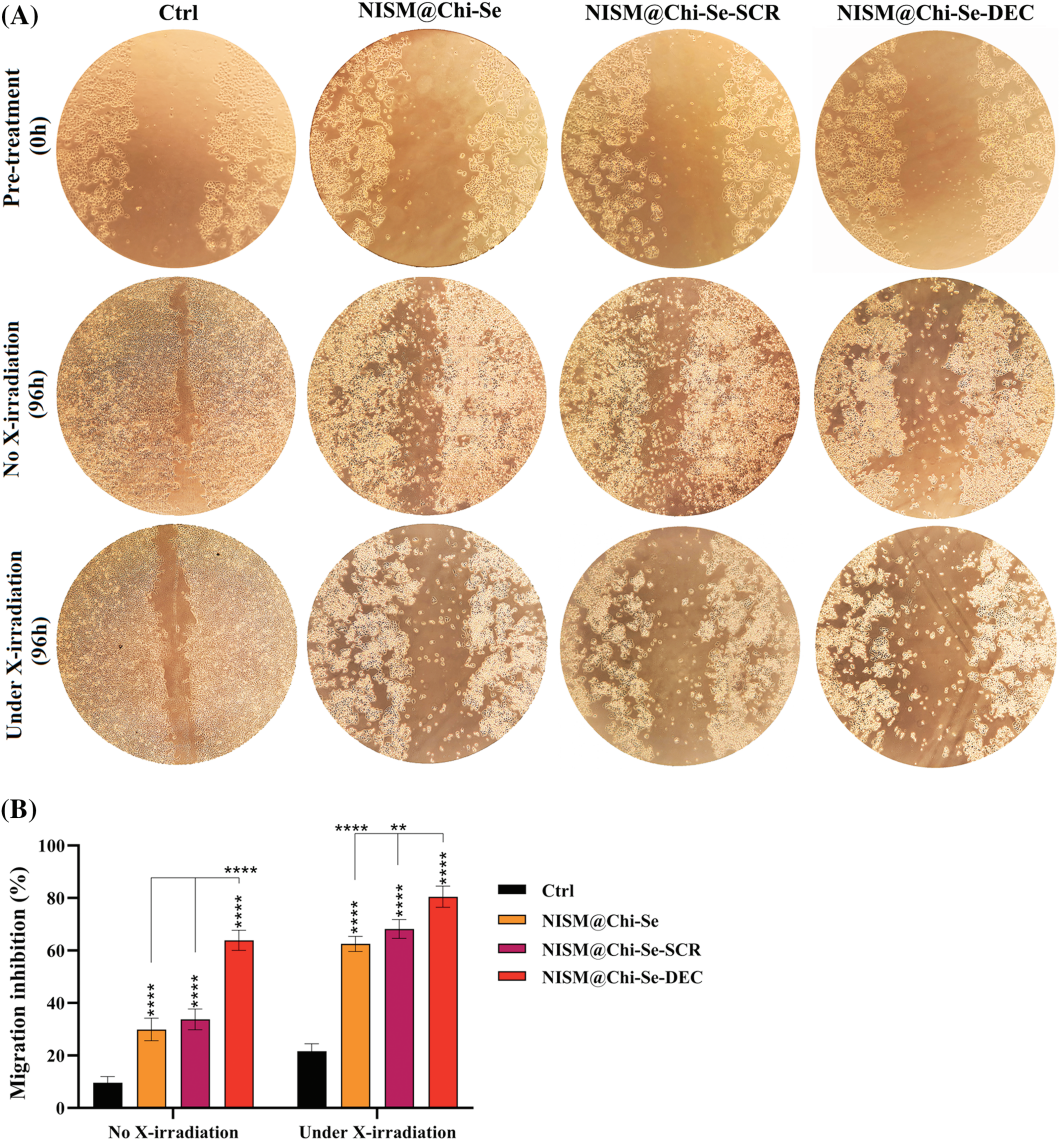
(A) Evaluation of scratch repair ability in MDA-MB-468 cells after treatment with 1 μg/mL from NISM@Chi-Se, NISM@Chi-Se-SCR, and NISM@Chi-Se-DEC nanocarriers in conditions with and without X-irradiation. (B) Statistical analysis of scratch repair rate in treated groups. **p < 0. 01 and ****p < 0.0001.

In the absence of X-ray exposure, the gap created in the control group was mainly filled, while scratch repair inhibition was observed in the groups treated with NISM@Chi-Se nanocarriers and NISM@Chi-Se-SCR. It was statistically significant compared to the control group. The induction of cell migration inhibition was significantly higher in the group treated with NISM@Chi-Se-DEC nanocarriers than in the other groups (Fig. 11).

Under X-ray exposure, inhibition of scratch repair (cell migration) occurred in all treated groups, with a significant difference compared to the control group. The maximum scratch repair inhibition was observed in the group treated with NISM@Chi-Se-DEC along with 2 Gy X-irradiation (Fig. 11).

## Discussion

New cancer therapeutic approaches, such as siRNA, miRNA, and oligodeoxynucleotide decoy (ODNs), have been proposed as alternatives to conventional treatments due to their effectiveness and fewer adverse effects [31]. Myc is a crucial oncoprotein that is overexpressed in 50%–70% of cancers, including breast cancer and is associated with immune escape, resistance to apoptosis, metastasis, and other cancer-promoting processes [32]. The decoy ODNs strategy can be used to inhibit Myc oncoproteins. In this study, we developed a nanocarrier system (NISM@Chi-Se-DEC) by loading decoy ODN against Myc TF (DEC) and selenium nanoparticles (SeNPs) onto chitosan (Chi) polymer, which was then encapsulated in niosomes. The combination of selenium and chitosan in the nanocarrier structure was aimed at enhancing the effect of X-ray irradiation and increasing encapsulation efficiency, respectively. X-ray irradiation was used in combination with NISM@Chi-Se-DEC in cancer cell treatment to provide the benefits of combination therapy.

The decoy ODN used in this study was designed from www.cisterome.org database, based on the consensus sequence “CACGTG” for the Myc binding site, as previously reported [24,33]. After synthesizing various groups of nanocarriers, their physicochemical properties were characterized.

The successful synthesis of the nanocarriers was examined using DLS and FT-IR techniques. The results revealed an increase in the hydrodynamic size of free NISM after encapsulation and modification with decoy ODNs, selenium, and chitosan. Furthermore, the *zeta* potential of NISM@Chi-DEC-Se was found to be positive, in contrast to the negative charge of free NISM, owing to the presence of chitosan. This positive charge is responsible for the high cellular uptake of the nanocarriers. The size of the nanocarriers is a crucial factor in drug delivery to cancerous tissues. Smaller nanocarriers with a size below 200 nm can easily penetrate tumor vessels due to the enhanced permeation and retention (EPR) effect. Lipid nanocarriers are generally suitable within the size range of 50–1000 nm. However, nanocarriers larger than 500 nm are not ideal for *in vivo* administration due to their rapid clearance from circulation. Accordingly, the size of NISM@Chi-DEC-Se nanocarriers is appropriate for *in vivo* studies [34]. FESEM was used to study the morphology and size of the nanocarriers. The results showed that NISM@Chi-Se-DEC has a spherical and regular shape, with a size of 59.75 nm. The FESEM size results were smaller than the DLS results because, unlike DLS, FESEM analyzes samples in dry form. Likewise, the FT-IR results showed that Se, DEC, and chitosan were successfully encapsulated in NISM, which was consistent with the previous studies [35].

The release behavior of decoy ODNs was studied at pH 5.8 and 7.4, which correspond to the endosomal and bloodstream pH levels, respectively. The results showed that in the NISM@Chi-Se-DEC group, approximately 50% of the ODNs were released after 144 h at pH 7.4, and about 60% of the ODNs were released after 144 h at pH 5.8. These findings suggest that the release of ODNs predominantly occurs in the intracellular endosome, rather than in the bloodstream. Moreover, the release of ODNs exhibits time-dependent behavior [36].

The results of hemolysis showed that NISM@Chi-Se-DEC caused only about 11% hemolysis at the highest tested concentration (200 mg/mL). Notably, selenium had the lowest percentage of hemolysis among the tested agents, which is consistent with its status as a microelement in the animal body. These findings indicate that NISM@Chi-Se-DEC is biocompatible and can be used for *in vivo* studies.

The uptake results showed that the cell control (Ctrl) and nanocarrier control (NISM@Chi-Se) groups had minimal uptake, while the uptake of NISM@Chi-Se-ODN (ODNs labeled with Cy3 fluorescent) was significantly higher. Additionally, NISM@Chi-Se-ODN exhibited dose-dependent uptake behavior. A partial amount of fluorescence was observed in the ctrl and NISM@Chi-Se groups, likely due to light emitted from the cell organelles and the nanocarriers, respectively.

Without X-ray exposure conditions, the MTT results showed that at a concentration of 0.5 µg/mL of nanocarriers, none of the treatment groups caused a significant difference in cytotoxicity. However, at a concentration of 4 μg/mL, the cytotoxicity in all treatment groups (except for selenium) significantly increased, indicating that the concentration of 4 μg/mL is toxic. At a concentration of 1 μg/mL of NISM@Chi-DEC and NISM@Chi-Se-DEC, the cytotoxicity was significantly higher in comparison to the control group. Moreover, the cytotoxicity in the NISM@Chi-Se-DEC group was greater than that of the NISM@Chi-DEC group, which could be attributed to the presence of selenium. Selenium is known to be one of the essential elements for cell growth, and at low doses, it can increase cell growth. This finding is consistent with the study conducted by Pekarkova et al., which evaluated the effect of selenium on cell viability [37]. Moreover, a significant increase in cytotoxicity was observed at 1 μg/mL of NISM@Chi-Se-DEC compared to NISM@Chi-Se (free nanoparticles) and NISM@Chi-Se-SCR (ODN decoy negative control) at the same concentration. This finding indicates that the decoy oligodeoxynucleotide effectively inhibits the Myc oncoproteins and increases cytotoxicity. Based on the uptake and MTT results, a concentration of 1 μg/mL was selected for the subsequent tests.

In 2016, Yin et al. reported that exposure to X-rays leads to overexpression of Myc oncoproteins in triple-negative breast cancer cell lines [38]. As Myc is one of the essential oncoproteins in cancer cells and can lead to various harmful effects, such as resistance to apoptosis and metastasis, this finding highlights the importance of addressing this problem in cancer treatment. X-ray therapy (radiotherapy) is the primary conventional treatment for all cancer types. However, to enhance the efficacy of treatment, combination therapy is often necessary. Therefore, we evaluated the effect of simultaneous X-ray irradiation and Myc inhibition in triple-negative breast cancer.

When the MTT test was performed under X-ray exposure conditions, a significant increase in cytotoxicity was observed in groups of nanocarriers that contained selenium in their structure. Specifically, significant increases in cytotoxicity were observed in the SeNPs, NISM@Chi-Se, NISM@Chi-Se-SCR, and NISM@Chi-Se-DEC groups at all concentrations tested. Furthermore, a significant increase in cytotoxicity was observed at a concentration of 1 μg/mL of NISM@Chi-Se-DEC compared to a similar concentration of NISM@Chi-Se (as free nanoparticles) and NISM@Chi-Se-SCR (decoy ODN negative control). These findings are in line with the study conducted by Chen et al., who reported that combining selenium nanoparticles with X-rays enhanced the anticancer effects of radiation therapy [39].

The cell cycle assay results showed that treatment with NISM@Chi-DEC and NISM@Chi-Se-DEC significantly arrested MDA-MB-468 cells in the G1 phase. In contrast, treatment with culture medium (Ctrl), NISM@Chi-Se (as free nanocarriers), NISM@Chi-SCR, and NISM@Chi-Se-SCR (as negative controls for decoy ODN) resulted in more cells in the S-phase and fewer cells in the G1 phase. Similarly, a study by Jing et al. in 2016 reported that Myc inhibition in MDA-MB-468 causes cell arrest in the G1 phase [40].

Under X-ray irradiation conditions, cell cycle analysis showed that treatment with nanocarrier groups containing selenium in their structure (NISM@Chi-Se, NISM@Chi-Se-SCR, and NISM@Chi-Se-DEC) led to cell arrest in the G2/M phase in MDA-MB-468 cells. Notably, the main G2/M arrest was observed in the NISM@Chi-Se-DEC group, which may be attributed to the simultaneous effect of Myc inhibition with decoy ODNs and the X-ray enhancement effect with selenium nanoparticles. Similarly, Chen et al. investigated the effect of combining selenium nanoparticles with X-ray irradiation on a breast cancer cell line. According to their study, a combination of 6 Gy X-ray irradiation with 0.3 μg/mL of selenium nanoparticles caused 48% of MCF7 cells to arrest in the G2/M phase [39].

The apoptosis results showed that in the absence of X-ray irradiation, the aggregation of apoptosis and necrosis in the NISM@Chi-DEC and NISM@Chi-Se-DEC groups were approximately 15% and 14%, respectively. Moreover, the rate of apoptosis in these two groups was significantly different from those of the control group (Ctrl) and the NISM@Chi-Se group (free nanocarriers). In cancer cells, Myc increases cell viability by overexpressing Bcl2 and NF-κB. Therefore, when Myc is inhibited by decoy ODN, the levels of Bcl2 and NF-κB are reduced, leading to the activation of apoptotic pathways in the cell [41].

In the apoptosis test under X-ray irradiation conditions, the induction of apoptosis in all treatment groups was significantly different from that in the cellular control (Ctrl). This may be due to the high sensitivity of cells after X-irradiation. Moreover, NISM@Chi-Se-DEC caused a significant increase in apoptosis compared to the control (Ctrl), NISM@Chi-Se (free nanocarriers), and NISM@Chi-DEC groups, demonstrating the effect of combined therapy, i.e., simultaneous Myc inhibition effect with decoy ODNs and X-ray enhancement effect with selenium nanoparticles. Similarly, in 2013, Lee et al. investigated the anticancer effects of simultaneous co-inhibition of epidermal growth factor and insulin-like growth factor-1 receptor in combination with X-ray irradiation. The apoptosis assay in aforementioned study was performed with 10 μM AG1478 and 10 μM AG1024 in combination with 4 Gy X-ray irradiation on the MDA-MB-468 cell line. The results showed that the apoptosis rate in MDA-MB-468 cells was increased when the treatment agents were used in combination with X-ray radiation [42].

In cancer cells, the transcription factor Myc causes overexpression of miRNA-186 and miR-9, which inhibit E-cadherin, as well as PRS-19, while inhibiting NDRG2 expression, ultimately leading to epithelial-mesenchymal transition (EMT) in the cancer cell [43]. Our scratch results showed that Myc inhibition caused migration inhibition. Moreover, the combination of ODNs decoy against Myc with X-irradiation enhanced the cell migration inhibition. Indeed, NISM@Chi-Se-DEC inhibited MDA-MB-468 migration under X-ray exposure.

## Conclusion

We successfully synthesized NISM@Chi-Se-DEC, a hybrid nanocarrier containing Myc decoy ODN, to investigate the potential of combination therapy for triple-negative breast cancer. NISM@Chi-Se-DEC showed enhanced cellular uptake, increased cytotoxicity, induced apoptosis, arrested cell cycle, and inhibited cellular migration under X-ray exposure. This suggests that treatment of TNBC cells with NISM@Chi-Se-DEC nanostructure along with radiotherapy can increase the sensitivity of cancer cells toward X-irradiation which may be a promising approach for treating triple-negative breast cancer. Our future research will be on investigating the anticancer effects of this nanostructure *in vivo* studies with a focus on challenges such as modifying the niosomes nanocarriers to reduce carrier toxicity and improve active targeting to tumor cells.

## Supplementary Materials

Figure S1 Percentage of hemolysis of NISM, NISM@Chi, NISM@Chi-Se, NISM Chi-SCR, NISM@Chi-DEC, NISM@Chi-Se-SCR, NISM@Chi-Se-DEC and SeNPs at concentrations of 6.25, 12.5, 25, 50, 100, and 200 mg/mL. *p* <0.05 (*).

Figure S2 (A) Evaluation of cell population distribution in the cell cycle of MDA-MB-468 cells after treatment with NISM@Chi-Se, NISM@Chi-SCR, NISM@Chi-DEC, NISM@Chi-Se-SCR and NISM@Chi-Se-DEC with a concentration of 1 μg/mL in no X-irradiation conditions (B) Statistical analysis graph. *p* <0.05 (*), *p* <0. 01 (**), *p* <0.001 (***), and *p* <0.0001 (****).

Figure S3 (A) Apoptosis evaluation without X-irradiation conditions in MDA-MB-468 cells 24 h after treatment with culture medium (Ctrl), NISM@Chi-Se, NISM@Chi-SCR, NISM@Chi-DEC, NISM@Chi-Se-SCR and NISM@Chi-Se-DEC groups with a concentration of 1 μg/mL. (B) The column graph shows the apoptosis rate analysis following nanocarriers treatment. *p* <0.05 (*), *p* <0. 01 (**), *p* <0.001 (***), and *p* <0.0001 (****).

## Data Availability

The data sets used and/or analyzed during the current study are available from the corresponding author upon reasonable request.

## List of Abbreviations

SeNPs: Selenium nanoparticles
BSA: Bovine serum albumin
Chi: Chitosan
BC: Breast cancer
BCSCs: Breast cancer stem cells
TNBC: Triple-negative breast cancer
EDC: 1-(3-dimethylaminopropyl)-3-ethylcarbodiimide hydrochloride
NHS: N-hydroxysuccinimide
Cy3: Cyanine-3
Se: Selenium
ODNs: Oligodeoxynucleotides
SCR: Scrambled
TFD: Transcription factor decoy
PS: Phosphorothioate
PDI: Polydispersity Index
DLS: Dynamic light scattering
FT-IR: Fourier transform infrared
TEM: Transmission electron microscope
SDS: Sodium dodecyl sulfate
PI: Propidium iodide

## Supplementary Materials

The supplemental material for this article is available online at https://doi.org/10.32604/or.2023.043576.
