# Supplementary figures and images for: Combinational therapy with Myc decoy oligodeoxynucleotides encapsulated in nanocarrier and X-irradiation on breast cancer cells
Source: Oncol Res. 2023 Dec 28;32(2):309–23. doi: 10.32604/or.2023.043576 (PMC10765119; doi:10.32604/or.2023.043576)

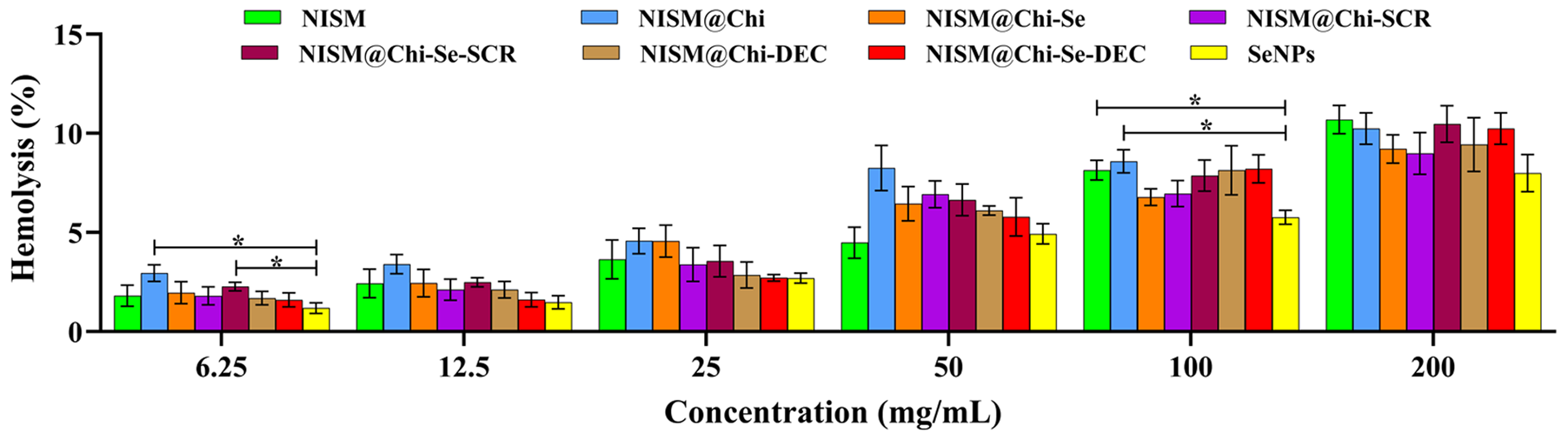

Supplement: Figure S1 [file OncolRes-32-43576-s001.tif]

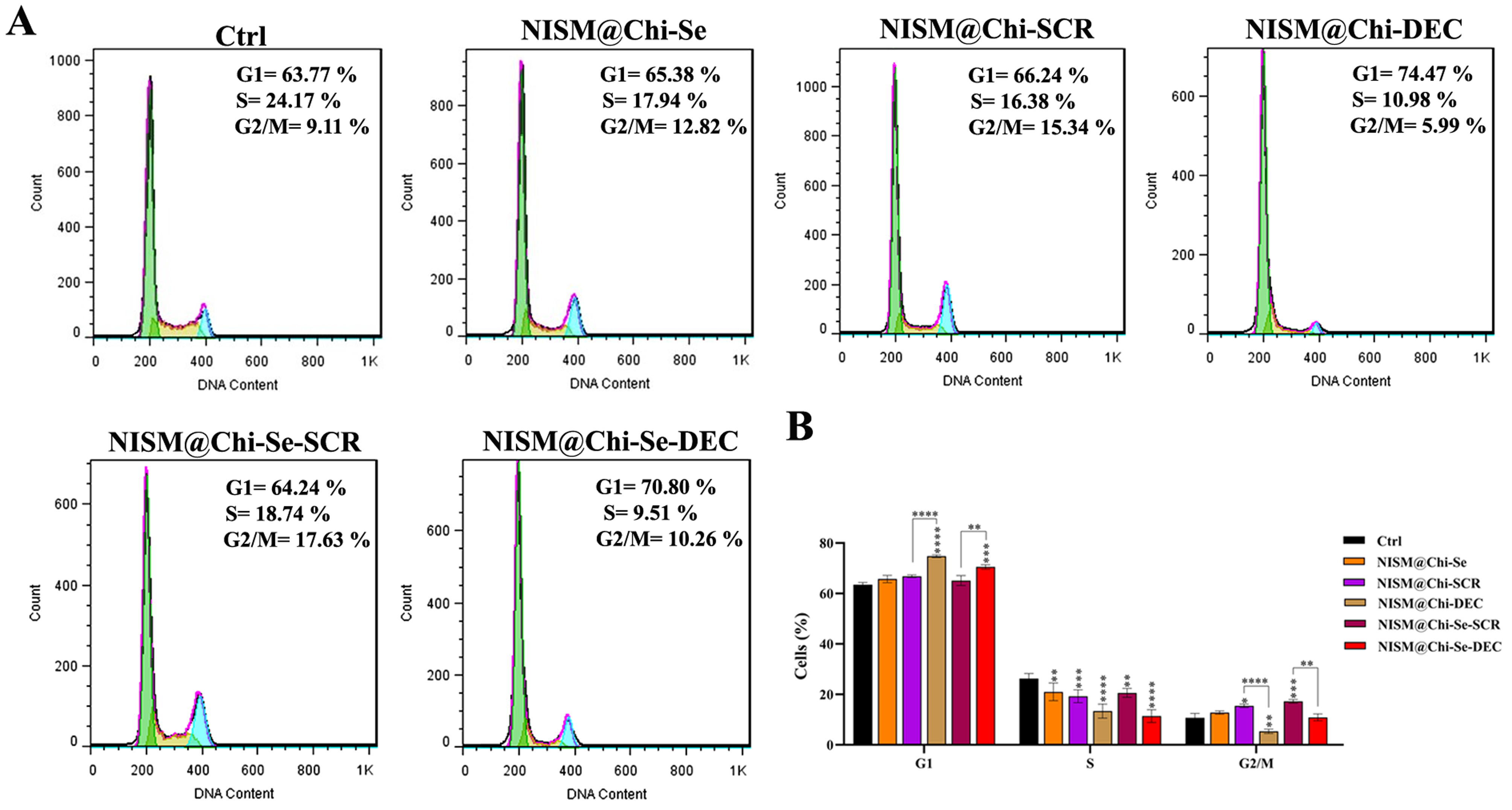

Supplement: Figure S2 [file OncolRes-32-43576-s002.tif]

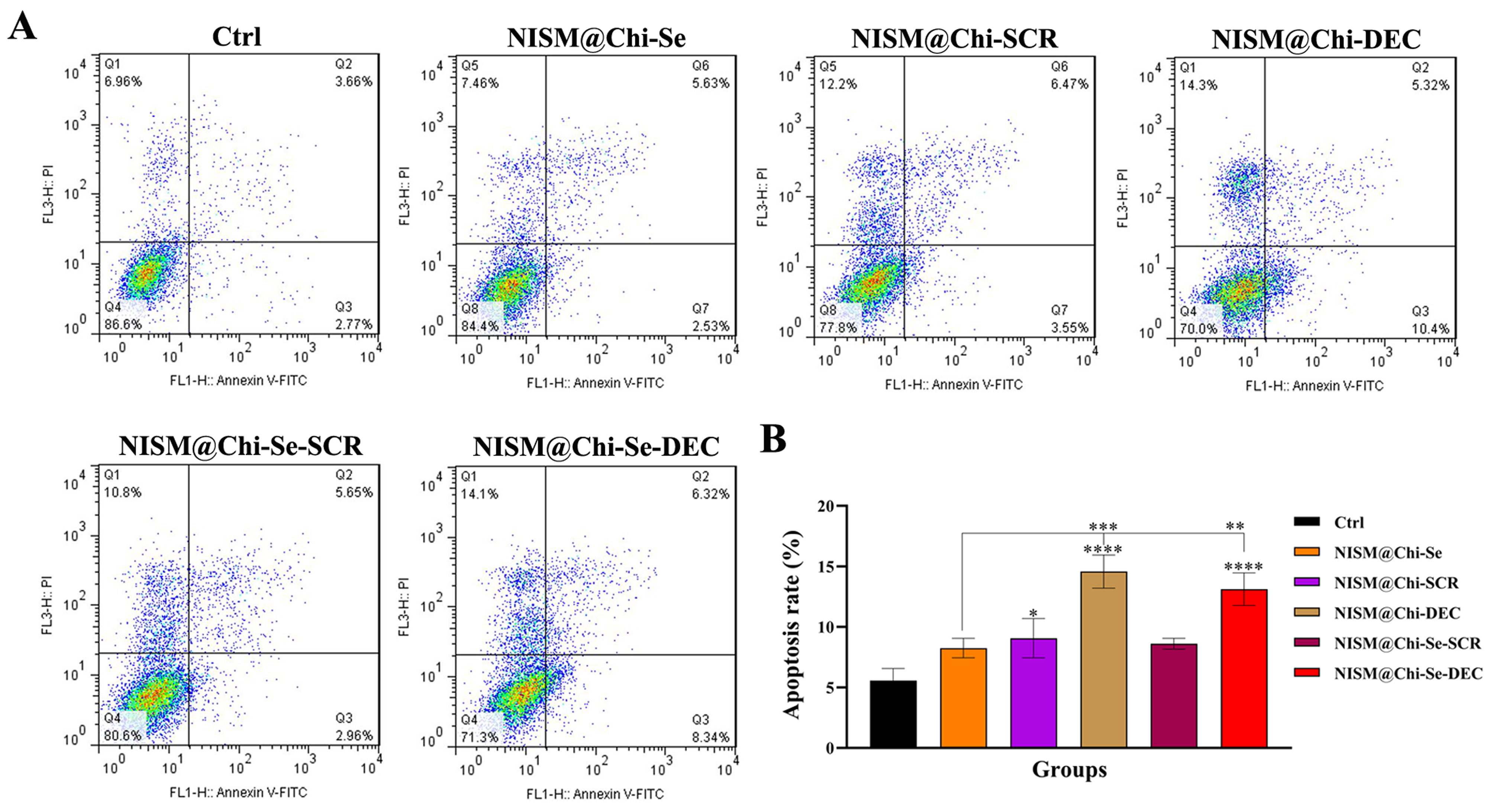

Supplement: Figure S3 [file OncolRes-32-43576-s003.tif]
